# Prioritization of Managed Pork Supply Movements during a FMD Outbreak in the US

**DOI:** 10.3389/fvets.2016.00097

**Published:** 2016-10-31

**Authors:** Gilbert R. Patterson, Alicia H. Mohr, Tim P. Snider, Thomas A. Lindsay, Peter R. Davies, Tim J. Goldsmith, Fernando Sampedro

**Affiliations:** ^1^Center for Animal Health and Food Safety, College of Veterinary Medicine, University of Minnesota, St. Paul, MN, USA; ^2^Research Support Services, College of Liberal Arts LATIS, University of Minnesota, Minneapolis, MN, USA; ^3^PIC, Hendersonville, TN, USA; ^4^Veterinary Population Medicine, College of Veterinary Medicine, St. Paul, MN, USA

**Keywords:** swine, risk prioritization, business continuity, movement restrictions, FMD

## Abstract

In the event of a foot-and-mouth disease (FMD) outbreak in the United States, local, state, and federal authorities will implement a foreign animal disease emergency response plan restricting the pork supply chain movements and likely disrupting the continuity of the swine industry business. To minimize disruptions of the food supply while providing an effective response in an outbreak, it is necessary to have proactive measures in place to ensure minimal disease spread and maximum continuation of business. Therefore, it is critical to identify candidate movements for proactive risk assessments: those that are both most likely to contribute to disease spread and most necessary for business continuity. To do this, experts from production, harvest, retail, and allied pork industries assessed 30 common pork supply movements for risk of disease spread and industry criticality. The highest priority movements for conducting a risk assessment included the movement of weaned pigs originating from multiple sow farm sources to an off-site nursery or wean to finish facility, the movement of employees or commercial crews, the movement of vaccination crews, the movement of dedicated livestock hauling trucks, and the movement of commercial crews such as manure haulers and feed trucks onto, off, or between sites. These critical movements, along with several others identified in this study, will provide an initial guide for prioritization of risk management efforts and resources to be better prepared in the event of a FMD outbreak in the United States. By specifically and proactively targeting movements that experts agree are likely to spread the disease and are critical to the continuity of business operations, potentially catastrophic consequences in the event of an outbreak can be limited.

## Introduction

In the event of a foot-and-mouse disease (FMD) outbreak in the United States., local, state, and federal authorities will implement an emergency response plan as described in the United States Department of Agriculture, Animal and Plant Health Inspection Service (USDA APHIS) Foreign Animal Disease Preparedness and Response Plan (1). This response includes a control and eradication strategy that will utilize depopulation, quarantine, vaccination, and managed movement control measures applied throughout the swine industry. The document recognizes the need to develop a strategic plan to address managed movement control and its implications for continuity of business in foreign animal disease preparedness planning (1).

Continuity of business, in the context of the food supply, means the ability of a farm or food processor to continue key operations of production and distribution of safe, high quality food, and agricultural commodities despite disruption of normal operational procedures (1, 2). These key operations are critical to business vitality and may cause severe economic losses for the industry if disrupted for prolonged periods of time by managed movement controls (3). In order for any managed movement to take place, incident commanders must issue official movement permits for animals or commodities that have an acceptable level of risk. These permits need to be guided by a risk assessment or science-based evaluation (2).

Completing a risk assessment in a timely manner during an outbreak is typically impractical and not conducive for the coordination of managed movement (4). Developing risk assessments requires significant time, thereby potentially delaying the movement of pigs or pork products that may represent negligible risk for disease spread. Throughout the swine industry, there is a heavy reliance on continuous movement of animals, and the timely delivery of animal feed, supplies, and products. Even brief disruptions in the supply of products or movement of animals can result in devastating economic losses as well as serious animal welfare concerns, as available inventory capacity is often limited (3, 5).

Risk assessments conducted proactively, before an outbreak, can identify mitigation strategies to reduce the potential for disease spread and facilitate business continuity. This is done by supporting the timely movement of animals and products that represent an acceptable low risk for disease spread, while providing additional resources and safeguards to restrict those movements that pose a high risk of spreading disease. It is this balance between “acceptable risk” of disease spread and importance for business continuity that incident commanders will be seeking when issuing managed movement orders. Invariably, there may exist movements that are both critical to business continuity, but also pose a high risk for disease spread. These movements are important candidates for conducting a proactive risk assessment due to the anticipated negative consequences for the overall industry if they are not completed in a timely manner. However, little information is available about which specific movements in the pork supply chain are critical to both the potential for disease spread and the economic viability of the industry. Identifying critical movements at the intersection of these two factors is essential for effectively guiding an emergency response in the face of a transboundary disease outbreak such as FMD in swine.

The objective of this study is to establish a framework for prioritizing critical movements within the pork supply chain according to experts’ perception of the likelihood of spreading FMD and the importance of the movements for the continuity of business.

## Materials and Methods

### Recruitment of Experts

To effectively evaluate the risk and impact of various movements in the pork supply chain, opinions were solicited from experts who were actively engaged within the swine industry, including pork producers, veterinarians, and academics. Experts were recruited from multiple parts of the production chain, so collectively they would be able to evaluate risk across all of the movements. An online survey (6) was distributed *via* email to the American Association of Swine Veterinarians (AASV) mailing list, and respondents were encouraged to forward the invitation to other industry professionals in an effort to capture a diversity of responses. AASV is a non-profit educational professional society for veterinarians that specialize in swine health and management for the purposes of pork production. To help recruit more experts, announcements about the survey were made at the 2015 Leman Swine Health Conference and at the 2015 World Pork Expo, which are two technical meetings attended by a large group of AASV members, as well as many other swine industry producers and professionals each year. The announcements were followed with an email and link to the survey in the weekly AASV e-newsletter.

Fifty-one experts completed the survey, and an additional 19 provided partial responses (a further 8 consented to participate but did not answer any question, so no information about them is known). Experts indicated their line of work in the survey, and respondents included swine producers (*n* = 9), harvest industry (*n* = 4), retail/distribution (*n* = 10), and allied industries (*n* = 47). Those in allied industries could specify one or more industries. Of those who specified (*n* = 31), responses included veterinarians (*n* = 25), non-veterinarian academic or government workers (*n* = 4), and media/industry (*n* = 2). Pork producers were asked additional demographic questions regarding the size of their production, the type of operation, and the frequency of pig movements. Producers and those in allied industries were also asked about the size and location of their facilities or location of their involvement, and whether they had biosecurity protocols in place.

### Prioritization of Critical Movements

To prioritize the critical movements among the pork supply chain, questions were included on the survey to elicit expert opinion on FMD-related threats with the goal of identifying movements that have the highest perceived risk of disease spread and that are understood to be most critical to the business operation. Thirty common pork supply movements were identified based on the structure of the current pork production chain in the United States (7). Included were movements of all live pigs, genetic material, feed, equipment, personnel, and materials that are common to the multistage production systems that predominate in the United States. Movements of finished pork products post-harvest were also considered. These movements were divided into five main categories of the pork production chain: equipment, genetics, general (live animals), harvest and processing, and personnel (Table 1).

**Table 1:** **Consensus scores for perceived risk of FMD spread, and the mean time until a negative impact on business continuity would occur for each movement**.

Category	Number	Consensus high risk of disease spread	Majority placement for risk of disease spread	Time to negative business impact	Movement description
Equipment and feed	1	59	Unclear consensus	2–7 days	Feed onto production sites
	2	54	Unclear consensus	7–14 days	Supplies onto production sites
	3	76	High	7–14 days	Shared equipment onto production sites
	4^a^	79	High	2–7 days	Contracted or shared livestock trucks onto production sites
	5	60	Unclear consensus	2–7 days	Dedicated livestock trucks among company production sites
Live animals	6	60	Unclear consensus	2–7 days	Weaned pigs to off-site nursery, wean to finish, or finishing (single source)
	7	78	High	7–14 days	Finishing pigs direct to slaughter
	8	69	Unclear consensus	14–21 days	Replacement gilts into a sow unit
	9^a^	90	High	2–7 days	Weaned pigs to off-site nursery, wean to finish, or finishing (multiple sources)
	10	65	Unclear consensus	7–14 days	Feeder pigs to finishing (e.g., from nursery to finishing)
	11	63	Unclear consensus	14–21 days	Cull sows and boars direct to slaughter
	12	84	High	14–21 days	Off size and cull pigs, sows, and boars to sale barn/buying station
	13^a^	80	High	7–14 days	Off size and cull pigs, sows, and boars from sale barn/buying station to slaughter
	14	82	High	14–21 days	Feeder pigs from sale barn to production site
	15	71	Unclear consensus	2–7 days	Dead stock to off-site disposal (landfill, rendering, etc.)
	16	55	Unclear consensus	14–21 days	Manure to field application off-site
Genetic	17	52	Unclear consensus	14–21 days	Replacement gilts and boars into production system isolation
	18	70	Unclear consensus	14–21 days	Replacement gilts and boars onto production site
	19	50	Unclear consensus	2–7 days	Semen into a production system (breeding herd)
Harvesting and processing	20	44	Unclear consensus	2–7 days	Fresh carcasses to off-site processing
	21	43	Unclear consensus	2–7 days	Raw inedibles (byproducts) from harvest site to further processing
	22	30	Unclear consensus	7–14 days	Rendered inedibles from harvest site to further processing
	23	19	Low	2–7 days	Finished products to distributing
	24	23	Low	2–7 days	Fresh products to point of service
	25	19	Low	7–14 days	Ready to eat products to point of service
Personnel	26^a^	76	High	2–7 days	Employees onto, off, and/or between production site(s)
	27	72	Unclear consensus	7–14 days	Routine service providers (e.g., plumbers, electricians, etc.) onto, off, and/or between sites
	28	67	Unclear consensus	7–14 days	Veterinarians onto, off, and/or between sites
	29^a^	82	High	7–14 days	Vaccination crews into, off, and/or between sites
	30^a^	95	High	7–14 days	Commercial crews onto, off, and/or between sites (e.g., manure haulers, feed trucks, and livestock haulers)

*^a^Indicates a priority movement identified in this study*.

Experts were asked to assign each of the thirty movements to one of the four categories describing its risk of disease spread: no or slight risk, low risk, some risk, or high risk of FMD disease spread. Then, they were asked to estimate the time at which the restriction of each movement during an outbreak would have a significant negative consequence on business (e.g., high likelihood of bankruptcy and negative impact on animal welfare). Time was expressed in a continuous scale using a slider with four labels from shortest (i.e., most critical, less than 48 h) to longest (i.e., least critical, more than 60 days). The slider was initially positioned at the longest time label. Respondents were instructed to assume all movements would take place according to their biosecurity protocols, if one existed, and to assume that movements were expected to occur the day after a restriction was implemented.

### Data Analysis

Perceptions of the risk of disease spread were assessed using an ordinal categorical scale (numeric values were never shown), so contiguous categories were not necessarily uniformly distanced from each other. Further, experts’ notion of the difference between any two contiguous choices could vary greatly. Therefore, it was not statistically appropriate to calculate means for this variable to assess overall perceptions of disease risk. Instead, these perceptions were analyzed by taking the upper two choices (“some risk” and “high risk”) as to indicate a substantial risk in the movement and the lower choices (“no or slight risk” or “low risk”) as to indicate no substantial risk. Therefore, scores were calculated based on the percent of experts who assigned a substantial risk for that movement (referred to as “high” for short from here on), with higher or lower percentages indicating a majority consensus. These “consensus” scores were used to identify the movements in which a substantial majority of experts (over 75%) agreed that the movement carries a high risk of disease spread. Conversely, a low percentage on these scores (below 25%) should be interpreted as a substantial consensus that a movement has low risk of disease spread, as it indicates the majority of experts determined the movement to have no or slight risk of disease spread.

**Figure 1:**
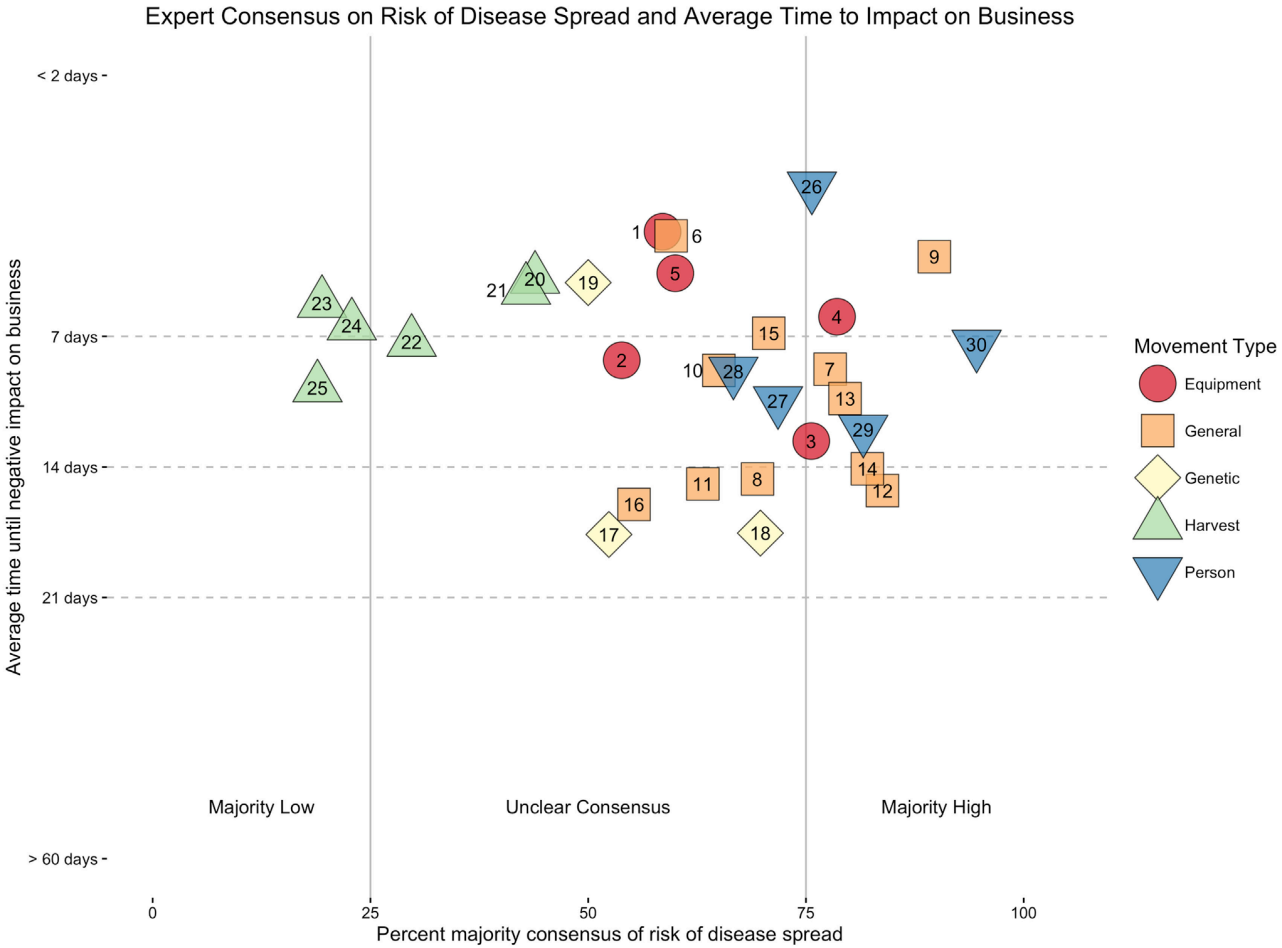
**Risk of disease spread consensus scores and mean time until a negative business impact (bars represent SEM) for each of the pork supply chain movements**. A specific description of each movement ID number can be found in Table 1. Detailed analysis of each movement, along with the precise breakdown of expert responses for each movement, is best appreciated in the interactive web-based version of this publication (http://z.umn.edu/pattersonfmd2016).

The other per-movement measure, time until critical business impact, was reported on a continuous slider scale, so the mean position of the sliders was calculated for each movement. However, because the labels placed on the scale were chosen to provide easily relatable time frames for respondents rather than to provide equidistant points on the timescale, absolute means of the positions were not a meaningful measure of time to critical business impact (e.g., a slider positioned two-thirds of the way between the labels >48 h and 7 days does not map onto an exact time value). For this reason, the mean times for each movement are reported as categories in Table 1, but are plotted based on their mean position in Figure 1 [for data and analysis scripts, see Ref. (6)].

Each movement was plotted based on the consensus scores of high risk of disease spread and the average time at which business would be critically impacted if the movement were stopped (Figure 1). To determine which movements would be best candidates for proactive risk assessments, individual movements were identified in which there was at least a 75% consensus of a high risk of disease spread, and a critical (time-sensitive) importance to business continuity, defined as negative business impact within 7 days (appearing in the top-right quadrant of the plot). Conversely, movements that were deemed by experts to have a low or negligible risk of disease spread, combined with a minimal impact on business continuity (lower left quadrant) were also identified.

## Results and Discussion

The recruited experts worked or owned facilities in many areas across the country. Of those who answered the demographic questions, 8 out of 10 producers and harvest industry respondents reported to own or manage farms or production facilities in multiple states, and most respondents in allied industries (34 out of 41; 82%) reported involvement in multiple states. Respondents also reported to work in each of the seven regions of the contiguous 48 United States. The vast majority of respondents reported having established biosecurity protocols for live animal sites or visits (42 out of 50; 84%).

When only considering the risk of disease spread, there were 10 movements in which most of the experts (greater than 75%) indicated that the movement had some or high risk of disease spread. The movements with the highest consensus of high risk of disease spread, in order of agreement, are (1) commercial crews onto, off, and/or between sites (95%); (2) weaned pigs to off-site nursery, wean to finish, or finishing (multiple sources) (90%); (3) off size and cull pigs, sows, and boars to sale barn/buying station (84%); (4) feeder pigs from sale barn to production site (82%); and (5) vaccination crews into, off, and/or between sites (82%; see Table 1). These results are consistent with current Food and Agriculture Organization of the United Nations (FAO) guidelines for animal disease risk management. According to FAO, animal diseases are spread most often by: the movements of live animals and animal products; by the transport of fomites, people, and equipment between farms; and animal comingling areas such as sale barns and slaughter plants (8).

When only considering impact on business, there were eight movements for which the mean of experts’ scores indicated business would be severely affected within 1 week of restriction. The movements with the shortest mean reported time to critical business impact were (1) employees onto, off, and/or between production site(s), (2) feed onto production sites, and (3) weaned pigs to an off-site nursery, wean to finish, or finishing (single source) (see Figure 1 and Table 1).

These results are consistent with the study conducted by Bargen and Whiting (5), which determined the time sensitivity of weaned pig movement off of sow farms. This was done by studying 15 sow farms in the Manitoba province of Canada and determined that if weaned pig movement off-site is restricted, the time to critical overcrowding was approximately 5 days (0.66 ± 0.88 weeks). Also, it makes logical sense that those movements pertaining to the basic husbandry of swine (feed availability, water, and environmental management), which are overseen by daily chore personnel, will have serious implications for animal health and welfare if disrupted (9).

Table 1 shows consensus scores for perceived risk of FMD disease spread, along with the mean time until a negative impact on business would occur across all 30 movement types. Movements with at least a 75% consensus of a high risk of disease spread, in combination with a time-sensitive window (less than 14 days) were defined as priority movements, in which proactive risk assessments would be most advantageous. There were three movements that met the criteria with the shortest time-sensitive window of 2–7 days. They were, in order of the highest percentage consensus for risk of disease spread: (1) weaned pigs to off-site nursery, wean to finish, or finishing (multiple sources), (2) contracted or shared livestock trucks onto, off, and/or between sites production sites, and (3) employees onto, off, and/or between sites. There were two additional priority movements that also had at least a 80% consensus for high risk of disease spread, however, had a longer time-sensitive window (7–14 days). These were, in order of the highest percentage consensus: (4) commercial crews onto, off, and/or between sites (e.g., manure haulers, feed trucks, and livestock haulers); (5) vaccination crews into, off, and/or between sites; and Off size and cull pigs, sows, and boars from sale barn/buying station to slaughter.

Returning to the movement of top priority, there are a number of features of moving weaned pigs to an off-site barn from multiple sources that may have prompted the experts to identify this particular movement as highest priority. There are a number of long-term health and logistical benefits that are captured when a barn is filled quickly with pigs of a similar age, which often requires inputs from multiple sow farm sources (9, 10). The nature of this movement, which may involve a trailer carrying weaned pigs to stop and pick additional pigs at multiple farms before arriving at its final destination, carries a risk of spreading disease to the farms visited. Additionally, when FMD virus naive pigs are presumably mixed with infected pigs at the destination site, virus spread is amplified in the new hosts, which will complicate further containment efforts (3).

Conversely, the movement of weaned pigs off a sow farm is a regular and essential function within the pork supply chain. In most cases, there is limited space available on sow farms to house weaned piglets for prolonged periods of time, and space must be made available frequently for the newest group of weaned pigs (5). For these reasons and according to the experts, this particular movement would have the strongest implications for the swine industry.

It is also noteworthy that movements related to the basic husbandry of swine, such as the movement of chore personnel and feed trucks, were widely considered to have a high risk for disease spread and of critical importance for business continuity. Movement restrictions that limit some of the basic needs of domestic swine (such as food and water) will unsurprisingly cause serious negative consequences if not tended to. This reality must be considered despite the high risk of spreading disease further. Previous studies on this topic have not examined the importance of personnel movement to provide basic animal husbandry, which highlights the need to consider these movements in national emergency preparedness plans.

Proactive risk assessments may also identify movements that should proceed in an FMD outbreak: those that that have a low risk of disease spread and would critically impact business in a short time if stopped. Two movements were perceived by experts to fall into this category. Less than 25% of experts identified “finished products to distributing” and “fresh products to point of service,” as having a high risk of disease spread. More specifically, 19% and 23% of experts, respectively, said the movements carried a high risk of disease spread, indicating the majority actually rated the disease spread as low. These movements were also perceived to critically impact business within 2–7 days, if stopped. This initial assessment would indicate that, in the event of an FMD outbreak, these two movements should be allowed to continue so as not to prevent finished products from reaching consumers and thus avoid interruption of the pork meat supply.

Conversely, there were no movements that were perceived by experts to have both a long time to critical business impact and a high risk of disease spread. However, two movements, which at least 75% of experts identified as having a high risk of disease spread, “off size and cull pigs, sows, and boars to sale barn/buying station” (84%) and “feeder pigs from sale barn to production site” (82%), were reported as having a time to critical business impact between 14 and 21 days. As described by Taylor and Rushton (8), sites where animals are comingled from multiple sources and subsequently transported back to another farm have the potential to spread the disease further. This combination of high perceived risk of disease spread and low time criticality may indicate that these movements are logical candidates for immediate restriction in the event of an FMD outbreak.

While this study provides an initial assessment of movements that would benefit from proactive risk assessments, there are several limitations that future research should address. First, the study did not assess the specific expertise or experience of respondents, which could have been used to weigh responses for given movements based on the level of familiarity/expertise each respondent had in each movement. Second, the expertise sample was predominantly veterinarians and those in allied industry. Future work should specifically target more experts from the producer and harvest industries.

## Conclusion

This work represents a preliminary descriptive analysis of the major pork supply chain movements, and the extent to which experts agree these movements may contribute to both FMD disease spread and how movement restrictions may critically impact business. While preemptive planning and risk assessment is underway to prepare for a potential FMD outbreak in the United States, it is important to consider whether the benefits of restricting movement (thereby reducing the size or duration of the outbreak) actually outweigh the costs (interrupting business continuity or causing animal welfare concerns). This analysis helps to provide some context for the determination of managed movements within the swine industry, while considering potential consequences of disease spread paired with time sensitivity.

A recent analysis conducted by Paarlberg et al. (11), on the potential cost of an FMD outbreak in the United States, across all livestock sectors, estimated a decrease of $14 billion (9.5%) in United States farm income. Losses in gross revenue for live swine were estimated at a 34% reduction, and pork products at a 24% reduction (11). Given the severe economic losses, which would result in the event of an FMD outbreak in the United States, it is important to consider options, which may help to limit the size and scope of an outbreak, as well as support the continuity of low-risk business operations in order to safeguard industry vitality.

To ensure its economic viability, the pork industry must place a high priority on the development of criteria and the facilitation of agreements to allow specific movements of live swine, industry personnel, and pork products during all phases and types an FMD outbreak. This work provides an initial step to guide emergency planning, as it reveals movements that are critical to business vitality, and should thus be the primary focus of proactive risk assessments in order to minimize disruption of these movements.

As these results show movements pertaining to basic swine husbandry as well as the movement of weaned pigs off of sow farms, pose both a high risk of disease spread paired with a short window of time before severe economic or animal welfare concerns are realized. Effectively managing these movements will therefore require careful consideration of the cost to benefit ratio when issuing movement permits. The information of this study can also be used to help determine which movements are of little consequence if they are temporarily restricted in an effort to contain the outbreak (such as the movement of cull animals), as well as those that would have severe economic consequences without contributing much to the containment of disease spread were they to be restricted (movement of pork products to the consumers).

## Author Contributions

GP – primary author. FS – contributing author. AM and TL – survey design and data analysis. TS, PD, and TG – contributing authors.

## Conflict of Interest Statement

The authors declare that the research was conducted in the absence of any commercial or financial relationships that could be construed as a potential conflict of interest.
